# *Veillonellaceae* family members uniquely alter the cervical metabolic microenvironment in a human three-dimensional epithelial model

**DOI:** 10.1038/s41522-021-00229-0

**Published:** 2021-07-06

**Authors:** Mary E. Salliss, Jason D. Maarsingh, Camryn Garza, Paweł Łaniewski, Melissa M. Herbst-Kralovetz

**Affiliations:** 1 Department of Obstetrics and Gynecology, College of Medicine-Phoenix, University of Arizona, Phoenix, AZ USA; 2 Department of Biology and Biochemistry, University of Bath, Bath, UK; 3 Department of Basic Medical Sciences, College of Medicine-Phoenix, University of Arizona, Phoenix, AZ USA; 4 Arizona State University, Tempe, AZ USA

**Keywords:** Antimicrobials, Microbiome, Biofilms, Cellular microbiology, Bacteriology

## Abstract

Bacterial vaginosis (BV) is a gynecologic disorder characterized by a shift in cervicovaginal microbiota from *Lactobacillus* spp. dominance to a polymicrobial biofilm composed of diverse anaerobes. We utilized a well-characterized human three-dimensional cervical epithelial cell model in conjunction with untargeted metabolomics and immunoproteomics analyses to determine the immunometabolic contribution of three members of the *Veillonellaceae* family: *Veillonella atypica*, *Veillonella montpellierensis* and *Megasphaera micronuciformis* at this site. We found that *Veillonella* spp. infections induced significant elevation of polyamines. *M. micronuciformis* infections significantly increased soluble inflammatory mediators, induced moderate levels of cell cytotoxicity, and accumulation of cell membrane lipids relative to *Veillonella* spp. Notably, both *V. atypica* and *V. montpellierensis* infections resulted in consumption of lactate, a key metabolite linked to gynecologic and reproductive health. Collectively our approach and data provide unique insights into the specific contributions of *Veillonellaceae* members to the pathogenesis of BV and women’s health.

## Introduction

Human mucosae are colonized by diverse and dynamic bacterial communities that impact health and homeostasis or contribute to disease states, depending on the compositional nature of the communities^1^. In the cervicovaginal microenvironment, *Lactobacillus* dominance is associated with optimal reproductive health^2^. Lactic acid, a by-product of *Lactobacillus* fermentation, is an important metabolite in maintaining host defense and protecting the cervicovaginal mucosal epithelium from infection by sexually transmitted pathogens and opportunistic commensal bacteria^3^. Overgrowth of diverse anaerobic bacteria and depletion of *Lactobacillus* spp. is characteristic of bacterial vaginosis (BV) in the cervicovaginal microenvironment^4,5^. Our group and others have developed a hypothetical model suggesting that early colonizers, such as *Gardnerella vaginalis* and *Prevotella bivia*, establish biofilm scaffolds over the cervicovaginal epithelium and exhibit low pro-inflammatory properties^4,6^. This model proposes that as the biofilm matures, *Lactobacillus* spp. become less abundant and secondary colonizers associate with the biofilm, which results in inflammation^4^. As *Lactobacillus* spp. are depleted, BV-associated bacteria produce polyamines that result in increased pH^4^.

BV is a common and highly recurrent cervicovaginal disorder^7^. Additionally, BV has been associated with adverse obstetric and gynecologic outcomes, such as preterm birth, increased acquisition of sexually transmitted infections, and possibly gynecologic cancer^8–13^. It is therefore imperative to better understand the pathophysiological mechanisms of BV. Key bacteria that have been the focus of BV studies thus far include *G. vaginalis, P. bivia, Sneathia amnii* and *Atopobium vaginae*, although more studies are required in order to completely elucidate contributions of the cervicovaginal microbiome to women’s health^10,14,15^. However, other vaginal commensal bacteria and BV-associated bacteria have not been studied in detail and their roles in health or BV remain obscure. For example, members of the *Veillonellaceae* family, such as *Veillonella atypica, Veillonella montpellierensis* and *Megasphaera micronuciformis*, are understudied bacteria that have been isolated from the female reproductive tract^16,17^.

*V. atypica* is commonly isolated from the oral cavity and has been studied extensively at this anatomical site^18–22^. In the oral microbiome, *V. atypica* is considered a ‘bridging species’ that enables the colonization of middle and late colonizers to the oral plaque biofilm, with the aid of initial colonizing species, therefore *V. atypica* promotes oral biofilm development^23–25^. In addition, *V. montpellierensis*, another *Veillonella* species, has been isolated from patients suffering from endocarditis^26^ and acute otitis media infections^27^. Similar to *V. atypica, M. micronuciformis* has also been isolated from the oral and gut microbiomes and is implicated in gastric cancer and response to chemotherapy^28–30^.

*M. micronuciformis* and other *Megasphaera* spp. are typically isolated in relatively high frequency from patients with BV, along with *Atopobium, Leptotrichia/Sneathia,* and *Eggerthella*-like species^31^. In contrast, *Veillonella* spp. are isolated from women with and without BV and have been associated with elevated cervicovaginal pH^31–33^. *Veillonella* spp. are commonly isolated with *Lactobacillus* spp., *G. vaginalis,* and *Peptostreptococcus* spp^31,32^. Overall, *Veillonellaceae* family members have been isolated from women with BV^16,17,32,34,35^, but their contributions to BV pathogenesis and biofilm formation remain understudied. Although the *Veillonella* genus is broadly reported, species-level specification is lacking in sequencing datasets^31,32^. Therefore, we chose *V. atypica, V. montpellierensis*, and *M. micronuciformis* to represent *Veillonellaceae* family members present in the female genital tract to better understand their potential contributions to BV and the local microenvironment. The specific species and strains used in this study were isolated from the female genital tract of women with and without BV and are available from the BEI Resources, NIAID, NIH as part of the Human Microbiome Project.

The functional impact of *V. atypica, V. montpellierensis,* and *M. micronuciformis* on host response mechanisms in the human cervix, which can contribute to poor gynecologic and reproductive outcomes, has not been previously investigated. Therefore, we aimed to evaluate the immunometabolic contributions of *V. atypica, V. montpellierensis,* and *M. micronuciformis* in the cervical microenvironment to better understand the role of these species in BV and other sequalae. To achieve this, we used a robust and extensively characterized human three-dimensional (3-D) cervical epithelial cell model for the study of host-microbe interactions and infected the model with clinical isolates of *Veillonellaceae* family members, coupled with untargeted global metabolomics and immunoproteomics approaches. We chose to use a 3-D cervical cell model due to the clinical relevance of studying BV-associated bacteria in the context of the cervix as it relates to human papillomavirus (HPV), HIV, *Chlamydia trachomatis*, and *Neisseria gonorrhoeae* infections^36–39^. Additionally, *M. micronuciformis* has been isolated from women suffering from preterm birth^40–42^. *Veillonella* spp. have been implicated in secondary bacteremia in a pregnant woman, but the potential for *Veillonella* spp. to participate as opportunistic pathogens in the female reproductive tract is understudied^43^.

The 3-D cervical model is a robust system to study host-microbe interactions, as it accurately resembles ultrastructural features of the cervical tissue in vivo, such as apical and basal differentiation, tight junctions, microvilli, and more organotypic surfaces for the colonizing bacteria, and has been shown to recapitulate clinical findings^15,44–47^. Herein, we applied a reductionist approach and infected the 3-D cervical model with single bacterial species to determine the individual contributions of these bacteria to the cervicovaginal microbiome that are currently lacking. This knowledge is essential to inform future experiments that involve polymicrobial infections^44–47^.

## Results

### *M. micronuciformis* infections induce moderate cytotoxicity to cervical epithelial cells

**Fig. 1:**
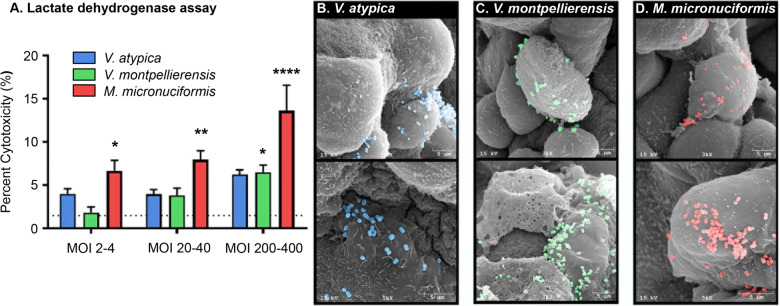
*M. micronuciformis* and *V. montpellierensis* induces significant cytotoxicity in 2-D cervical cell monolayers compared to *V. atypica*. **A** LDH assay to quantify cytotoxicity in cervical epithelial monolayers. *M. micronuciformis* infections induced significant cytotoxicity to cervical epithelial monolayers at a range of MOIs from 2 to 400. *V. montpellierensis* infections induced significant cytotoxicity at the highest MOI only. Error bars designate standard deviation. Statistical differences were tested using one-way ANOVA with Dunnett’s adjustment for multiple comparisons, *P < 0.05, **P < 0.005 ****P < 0.0001. Scanning electron microscopy (SEM) images of **B** *V. atypica*, **C** *V. montpellierensis*, and **D** *M. micronuciformis* colonizing 3-D cervical epithelial cells. SEM images were pseudo-colored using Photoshop 19.0 CC (Adobe).

First, we screened cervical epithelial monolayers for cytotoxicity following infection with *V. atypica, V. montpellierensis,* and *M. micronuciformis* at multiplicities of infection (MOIs) ranging from 2 to 400. We found that *M. micronuciformis* induced significant cytotoxicity at all tested MOIs (6.65%, 7.96%, and 13.63%) to cervical epithelial cell monolayers (*P* = 0.0291, *P* = 0.0041, and *P* < 0.0001 respectively); however, cervical epithelial cytotoxicity induced by *M. micronuciformis* infections was still modest at the highest MOI. *V. montpellierensis* infections induced cytotoxicity to cervical epithelial monolayers at only the highest MOI tested (6.50%, *P* = 0.0359). *V. montpellierensis* infections did not induce significant epithelial cytotoxicity at MOIs 2–4 and 20–40 (1.84%, 3.85%, respectively). Percentage cytotoxicity of *V. atypica* infections resulted in similar epithelial cytotoxicity at all tested MOIs (3.99%, 3.94%, and 6.23%) (Fig. 1A). Since *Veillonella* and *Megasphaera* spp. are predicted to harbor genes encoding lactate dehydrogenase (LDH)^48,49^, we also assessed the cytotoxicity using crystal violet staining (Supplementary Fig. 1). These results were consistent with LDH activity data with respect to cell viability measurements, indicating that potential bacterial LDH activity did not impact these data.

Next, we infected human 3-D cervical models with *V. atypica, V. montpellierensis,* and *M. micronuciformis* and confirmed colonization of the 3-D aggregates by SEM (Fig. 1B–D). Generally, *V. atypica, V. montpellierensis,* and *M. micronuciformis* sparsely colonized the 3-D cervical epithelial cells with occasional small clusters of bacteria co-localizing together on cervical cells. *V. montpellierensis* appeared to mostly colonize the surface of dead cells as visualized by SEM. *M. micronuciformis* also tended to sparsely cluster with other bacteria on cervical epithelial cell surfaces.

### Infection with *M. micronuciformis* induces significantly elevated 3-D cervical cell pro-inflammatory responses while *V. atypica* significantly decreases the expression of specific epithelial barrier targets

**Fig. 2:**
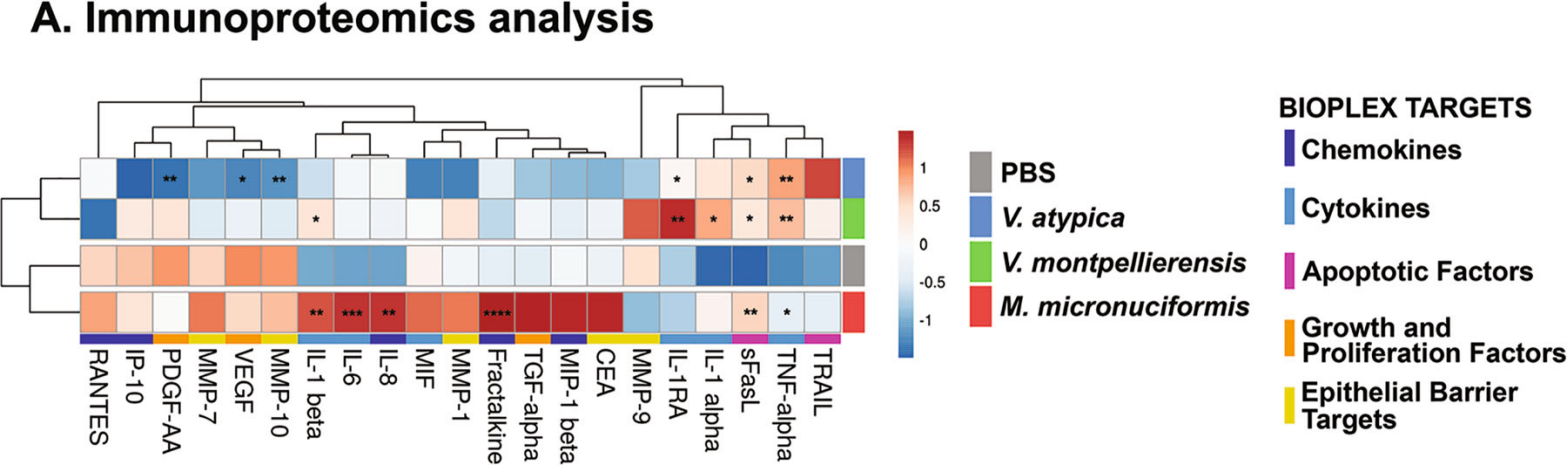
*M. micronuciformis* infections induced greater secretion of pro-inflammatory cytokines and chemokines, but *V. atypica* infections significantly decreased the culture concentration of some barrier targets. **A** Hierarchical clustering analysis (HCA) of immunoproteomics data from human 3-D cervical epithelial cells infected with *V. atypica, V. montpellierensis,* or *M. micronuciformis* under anaerobic conditions for 24 h. Statistical significance was determined using one-way ANOVA with Dunnett’s adjustment for multiple comparisons relative to PBS; *P < 0.05, **P < 0.01, ***P < 0.001, ****P < 0.0001. Color labels for each bacterial infection are observed to the right of the HCA and are as follows: PBS (gray), *V. atypica* (blue), *V. montpellierensis* (green), and *M. micronuciformis* (red). Protein targets (bottom of HCA) were color-coded in order to visualize clustering patterns of the protein targets: apoptotic factors (pink), epithelial barrier targets (yellow), growth and proliferation factors (orange), chemokines (dark blue), and cytokine (light blue). Individual concentrations can be visualized on graphs in the supplementary information (Supplementary Figs. 2–4).

Since BV is accompanied by mild genital inflammation, remodeling of the extracellular matrix, and cell lysis^46,50^, we examined the secretion of cytokines (IL-1α, IL-1β, IL-1RA, IL-6, and TNF-α), chemokines (fractalkine, IL-8, IP-10, MCP-1, MCP-3, MIP-1β, and RANTES), growth factors (PDGF-AA, TGF-α, and VEGF), apoptosis-related proteins (MIF, TRAIL, sFasL), mucins (CEA and CA125) and matrix metalloproteinases (MMP-1, MMP-7, MMP-9, MMP-10) in the 3-D cervical epithelial cell models infected with *V. atypica, V. montpellierensis* or *M. micronuciformis* at MOI 20–40 for 24 h. We analyzed 3-D cervical cell culture supernatants using cytometric bead arrays to quantify the host response to infection with these bacteria. *V. atypica* induced significant elevation of sFasL (*P* = 0.0202), IL-1RA (*P* = 0.0498), TNF-α (*P* = 0.0152), and significantly decreased secretion of PDGF-AA (*P* = 0.0083), VEGF (*P* = 0.0245), and MMP-10 (*P* = 0.0064). Infection with *V. montpellierensis* induced significant elevation of sFasL (*P* = 0.0250) and IL-1α (*P* = 0.0137), IL-1RA (*P* = 0.0095), TNF-α (*P* < 0.0001), IL-1β (*P* = 0.0470). *M. micronuciformis* significantly induced secretion of sFasL (*P* = 0.0065), TNF-α (*P* = 0.0056), IL-1β (*P* = 0.0012), IL-6 (*P* = 0.0004), fractalkine (*P* < 0.0001), and IL-8 (*P* = 0.0012) (Fig. 2A, Supplementary Figs 2–4).

We utilized hierarchical clustering analysis for evaluating immune response profiles for each bacterial infection*. V. atypica* and *V. montpellierensis* exhibited similar immunoproteomic profiles and clustered separately from *M. micronuciformis* or PBS controls (Fig. 2A). Overall, *M. micronuciformis* induced the secretion of pro-inflammatory cytokines and chemokines to a greater extent compared to *V. atypica* and *V. montpellierensis* and PBS controls. Infection with *V. atypica* resulted in a decrease in the concentration of PDGF-AA, VEGF, and MMP-10.

### Untargeted global metabolomics analysis revealed similar metabolic profiles between *V. atypica* and *V. montpellierensis* that are distinct from *M. micronuciformis*

**Fig. 3:**
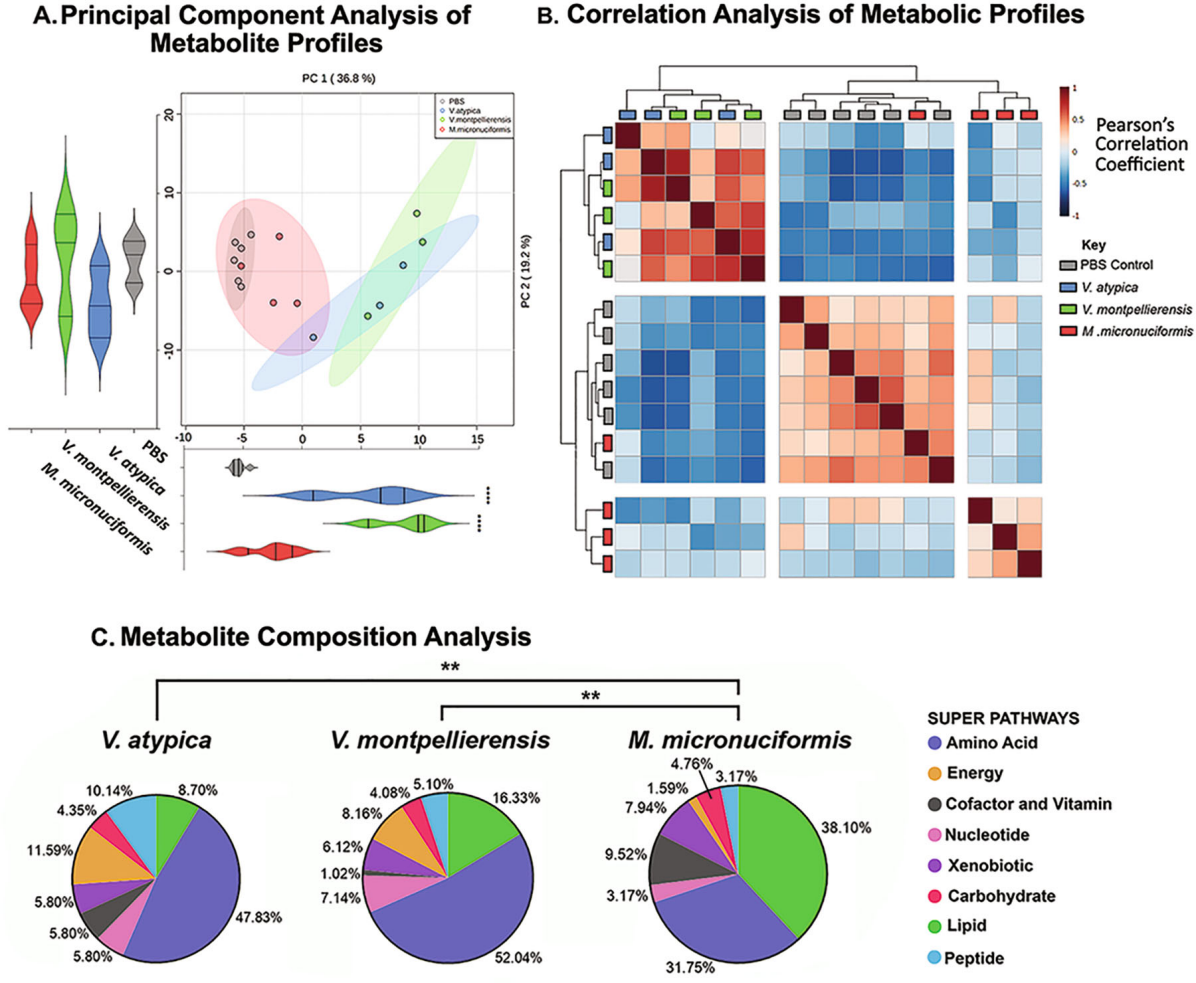
Both *Veillonella* species are metabolically similar to each other and distinct from PBS controls, while *M. micronuciformis* shares similar metabolic profiles with PBS controls. Global metabolic analysis of cell culture supernatants from human 3-D cervical epithelial cells infected with *V. atypica, V. montpellierensis,* or *M. micronuciformis* under anaerobic conditions for 24 h. **A** Principal component analysis (PCA) shading indicates the 95% confidence interval and **B** Pearson’s correlation analysis of metabolomics data displays how sample metabolic profiles relate to each other. Blue and red coloring indicate negative and positive correlations between samples, respectively. **C** Pie charts of significantly changed metabolites. The percentage of metabolites that were approached significance (*P* < 0.1) within each super pathway are labeled next to each slice (the total number of significantly altered metabolites for *V. atypica, V. montpellierensis,* and *M. micronuciformis* were 69, 98, 63 respectively). PCA significance (**A**) was tested by one-way ANOVA and Dunnett’s adjustment for multiple comparisons relative to PBS; ****P < 0.0001. The significance of differences between metabolite compositions (**C**) was tested using a chi-squared test of trends; **P < 0.01.

We next sought to define how *V. atypica, V. montpellierensis*, and *M. micronuciformis* alter the metabolomic landscape of 3-D cervical cell culture supernatants using high-throughput global untargeted metabolomics. Principal component analysis (PCA) of global metabolomic profiles (Fig. 3A) revealed that *V. atypica* and *V. montpellierensis* metabolomes cluster together, whereas *M. micronuciformis* and PBS controls tended to cluster together and separately from both *Veillonella* groups. Relative to PBS controls, PC1 (36.8% of explained variance) scores were significantly different between *V. atypica* and *V. montpellierensis* (*P* < 0.0001). No significant differences were found between PBS controls and any strain tested with respect to PC2 (19.2% of explained variance) scores.

We then applied non-parametric correlation analyses (Fig. 3B) to visualize the clustering of biological replicates. We observed that metabolomic profiles of *V. atypica* and *V. montpellierensis* correlated well with each other; however, the cluster analysis failed to group individual replicates of the same species, therefore suggesting highly similar metabolic profiles among the two *Veillonella* spp. While in the same parent cluster, the metabolomic profiles for *M. micronuciformis* and PBS controls mostly separated from each other. PBS controls correlated well between individual replicates, with the exception of one infection replicate with *M. micronuciformis*. Overall, the correlation analysis supported the PCA results.

We further investigated differences in metabolic profiles of *V. atypica, V. montpellierensis,* and *M. micronuciformis* infections by comparing the super-pathways of significantly changed metabolites relative to PBS controls (Fig. 3C). The composition of metabolic super-pathways for *M. micronuciformis* was significantly different from that of *V. atypica* (*P* = 0.0016) and *V. montpellierensis* (*P* < 0.0027). The super-pathways that were most different between *V. atypica, V. montpellierensis,* and *M. micronuciformis* were lipid (8.70%, 16.33%, and 38.1% respectively) and amino acid (47.83%, 52.04%, and 31.75% respectively).

### *Veillonella* spp. and *M. micronuciformis* infections result in enrichment in amino acids and lipids, respectively

**Fig. 4:**
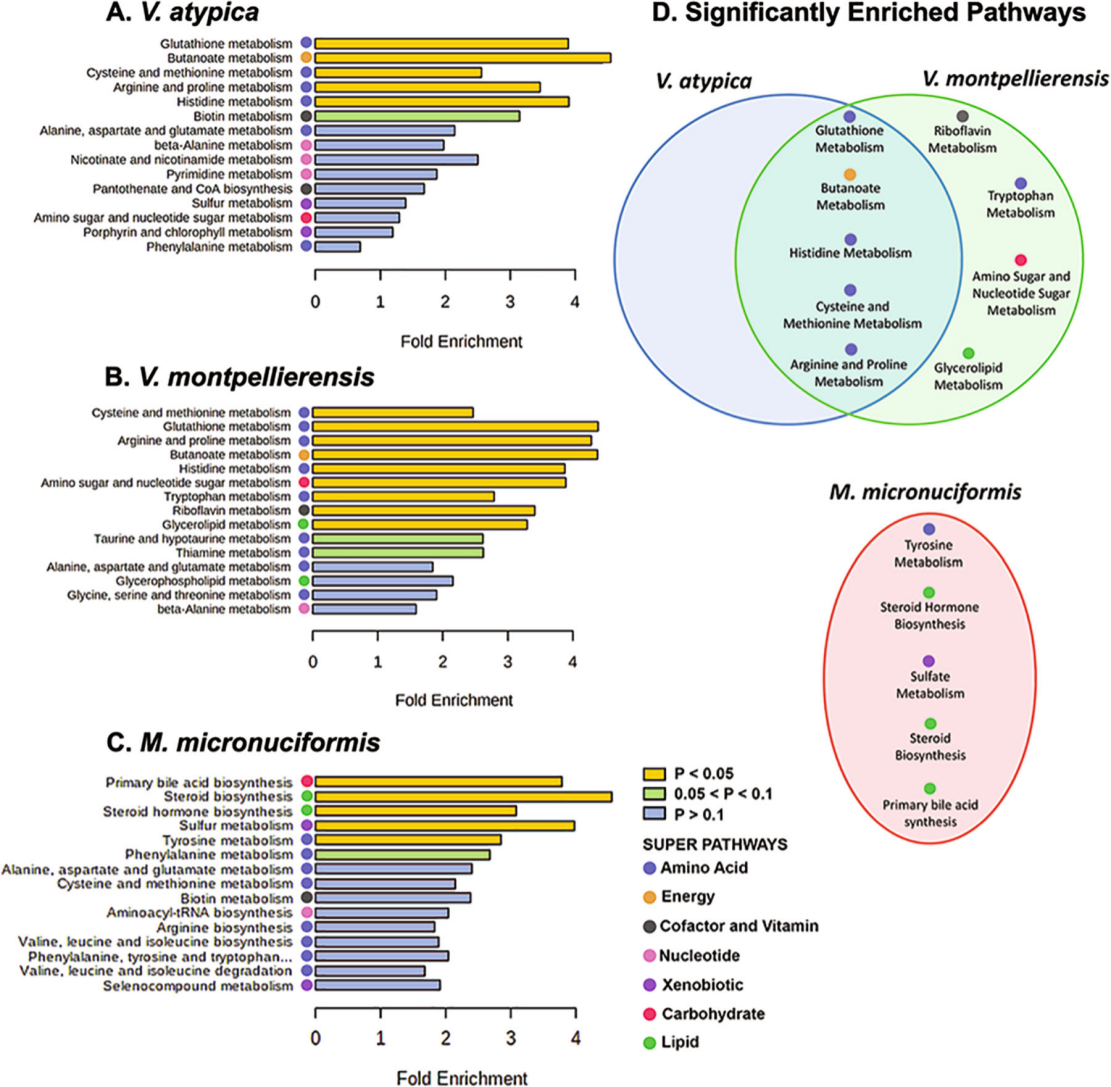
*V. atypica* and *V. montpellierensis* share overlapping enrichment of metabolic pathways belonging to amino acid metabolism that are distinct from *M. micronuciformis*. Metabolite pathway enrichment analysis of human 3-D cervical epithelial cells infected with **A** *V. atypica* (Supplementary Fig. 3), **B** *V. montpellierensis* (Supplementary Fig. 4), and **C** *M. micronuciformis* (Supplementary Fig. 5), under anaerobic conditions for 24 h. **D** Comparison of significantly enriched metabolic pathways between infections. Color bars indicate significance, yellow represents pathways that are significantly enriched (*P* < 0.05), green indicates pathways that approach statistical significance (0.05 < *P* < 0.1), and blue indicates pathways that were not significantly enriched (*P* > 0.1). The super pathway that a particular sub-pathway belongs to is indicated with colored circles.

Next, we performed metabolite pathway enrichment analysis using MetaboAnalyst (Fig. 3C). Metabolomic profiles from infection with *V. atypica* and *V. montpellierensis* were significantly enriched in metabolic pathways corresponding to arginine and proline (*P* = 0.0151 and *P* = 0.0076, respectively), butanoate (*P* = 0.0032 and *P* = 0.0060, respectively), cysteine and methionine (*P* = 0.0041 and *P* = 0.0014, respectively), histidine (*P* = 0.0106 and *P* = 0.0089, respectively) and glutathione (*P* = 0.0117 and *P* = 0.0061, respectively) metabolism (Fig. 4D). Infection with *V. montpellierensis* also significantly enriched four additional pathways corresponding to amino sugar and nucleotide sugar (*P* = 0.0199), riboflavin (*P* = 0.0425), tryptophan (*P* = 0.0394), and glycerolipid (*P* = 0.0497) metabolism.

Infection with *M. micronuciformis* modulated five significantly enriched pathways, including tyrosine metabolism (*P* = 0.0493), sulfate metabolism (*P* = 0.0309), steroid biosynthesis (*P* = 0.0159), steroid hormone biosynthesis (*P* = 0.0189), and primary bile acid synthesis (*P* = 0.01520). These pathways were unique to infection with *M. micronuciformis* relative to *V. atypica* or *V. montpellierensis* infections (Supplementary Figure 5).

We found variations in lipid metabolism both in our metabolite composition analysis (Fig. 3C) and enrichment analysis (Fig. 4D) for *V. montpellierensis* and *M. micronuciformis* infections. Significantly enriched pathways corresponding to lipid metabolism included glycerolipid metabolism and steroid and steroid hormone biosynthesis as modulated by *V. montpellierensis* and *M. micronuciformis*, respectively. Metabolic pathways corresponding to lipid metabolism were not significantly enriched by infection with *V. atypica*, therefore suggesting *V. montpellierensis* and *M. micronuciformis* influence species-specific lipid metabolic products following infection of 3-D cervical cells.

### *V. atypica* and *V. montpellierensis* infections resulted in significant elevation of polyamines and depletion of lactate, whereas glycerolipids were significantly elevated with *M. micronuciformis* infections

**Fig. 5:**
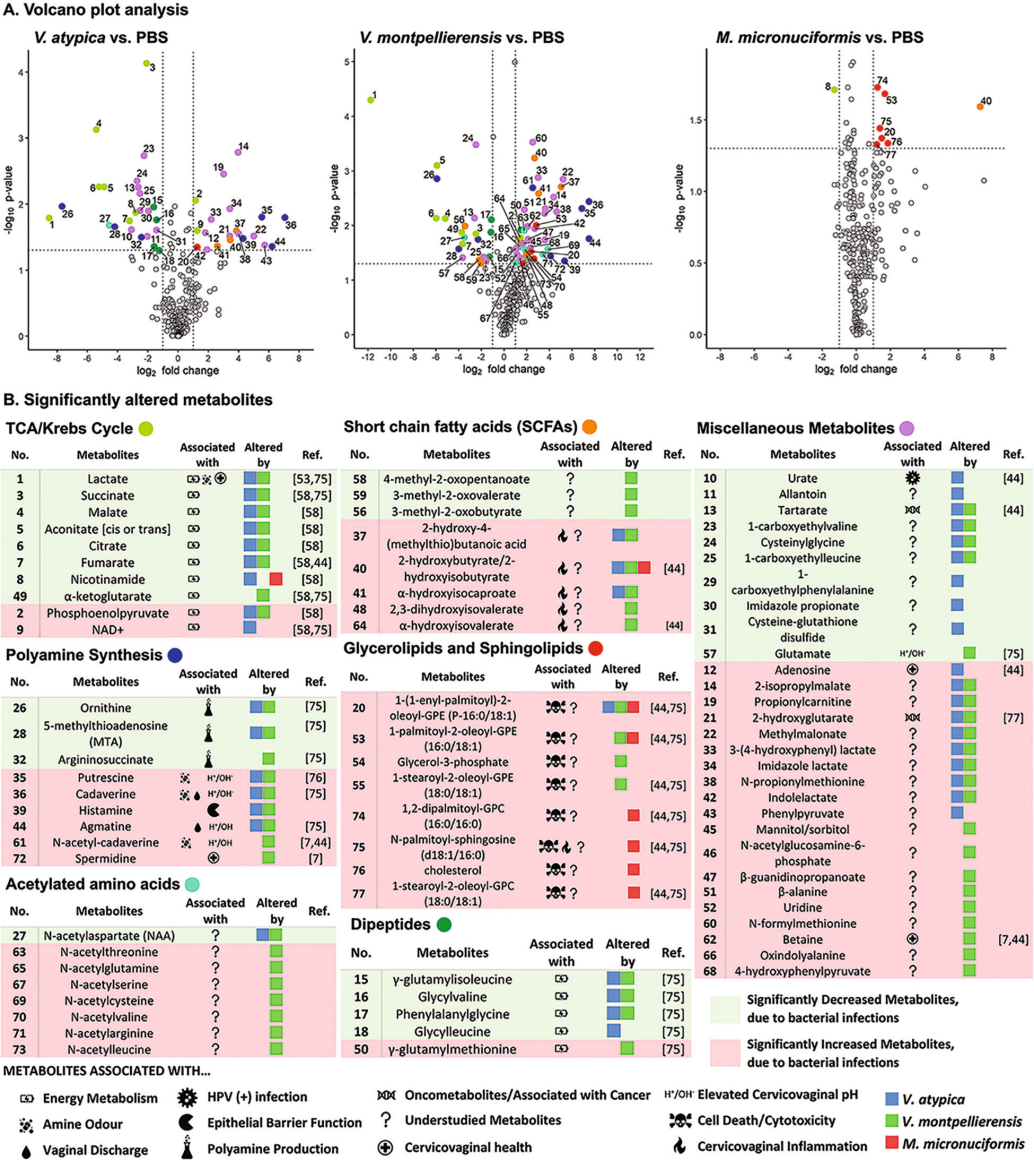
Metabolites associated with elevated cervicovaginal pH were significantly altered by *V. atypica* and *V. montpellierensis*, whereas metabolites associated with cell death were significantly accumulated with *M. micronuciformis* infections. Human 3-D cervical epithelial cells were infected with *V. atypica, V. montpellierensis,* or *M. micronuciformis* under anaerobic conditions for 24 h. **A** Volcano plots of *V. atypica*, *V. montpellierensis* and *M. micronuciformis* metabolic profiles. The *x* axis represents the log_2_ fold change values of metabolite scaled intensity values between the infectious treatment and PBS control. The *y* axis represents the −log_10_(*P*) value of differential metabolite abundance between infectious treatment and PBS controls (paired two-tailed Student’s *t*-test). **B** Significantly different metabolites (*P* < 0.05) with a fold-change < −2 or >2 are colored by super pathway. Symbols indicate how the metabolite may impact BV pathogenesis. See Supplementary Data 1 for specific *P*-values, *q*-values, and mean fold changes of all measured metabolites.

Finally, we assessed specific metabolites that were significantly elevated or depleted in response to *V. atypica, V. montpellierensis,* and *M. micronuciformis* infections relative to PBS controls. Culture supernatants from human 3-D cervical cells infected with *V. atypica* and *V. montpellierensis* significantly accumulated polyamines, such as cadaverine (*P* = 0.0159 and *P* = 0.0037), putrescine (*P* = 0.0155 and *P* = 0.00485), agmatine (*P* = 0.0444 and *P* = 0.0170), and histamine (*P* = 0.0331 and *P* = 0.0452) (Fig. 5B). Additionally, *V. montpellierensis* infections induced accumulation of spermidine (*P* = 0.0363) and *N*-acetyl-cadaverine (*P* = 0.00207). Metabolites involved in polyamine production were significantly depleted by infection with *V. atypica* and *V. montpellierensis*, including argininosuccinate (*P* = 0.0319 and *P* = 0.0179), ornithine (*P* = 0.0108 and *P* = 0.00140), and 5-methylthioadenosine (*P* = 0.0216 and *P* = 0.0266). The cervicovaginal pH-lowering metabolite, lactate, was also significantly depleted by infection with *V. atypica* and *V. montpellierensis* (*P* = 0.0162 and *P* < 0.0001, respectively).

Culture supernatants from 3-D cervical cells infected with *M. micronuciformis* accumulated glycolipids and sphingolipids (Fig. 5 and Supplementary Data 1). The short-chain fatty acid (SCFA) 2-hydroxybutyrate/2-hydroxyisobutyrate was also significantly elevated by infection with *V. atypica, V. montpellierensis,* and *M. micronuciformis* (*P* = 0.0344, *P* = 0.0006, and *P* = 0.0253, respectively). Specific glycerophospholipid and sphingolipid species were significantly elevated following *M. micronuciformis* infections including: 1,2-dipalmitoyl-GPC (16:0/16:0) (*P* = 0.0188), *N*-palmitoyl-sphingosine (d18:1/16:0) (*P* = 0.0363), cholesterol (*P* = 0.0465) and 1-stearoyl-2-oleoyl-GPC (18:0/18:1) (*P* = 0.0470). 1-(1-enyl-palmitoyl)-2-oleoyl-GPE (P-16:0/18:1) was significantly elevated by infection with *V. atypica, V. montpellierensis*, and *M. micronuciformis* (*P* = 0.0438, *P* = 0.0292, and *P* = 0.0426, respectively). In *V. montpellierensis* and *M. micronuciformis* infections, 1-palmitoyl-2-oleoyl-GPE (16:0/18:1) was significantly elevated (*P* = 0.00969 and *P* = 0.0209, respectively). Two glycerolipids were significantly elevated by infection with *V. montpellierensis*: glycerol-3-phosphate (*P* = 0.0405) and 1-stearoyl-2-oleoyl-GPE (18:0/18:1) (*P* = 0.0491). Overall, *M. micronuciformis* infections significantly elevated the glycerophospholipids and sphingolipids to a greater magnitude relative to *V. atypica* and *V. montpellierensis* infections.

In addition, 2-hydroxyglutarate (an oncometabolite) was significantly elevated during infection with *V. atypica* and *V. montpellierensis* (*P* = 0.0308 and *P* = 0.0052, respectively), with *V. atypica* infections (*P* = 0.0268), and betaine was altered with *V. montpellierensis* infections (*P* = 0.0102). Urate (an HPV-associated metabolite)^51^ was significantly increased with *V. atypica* infections (*P* = 0.0244).

Overall, infection of 3-D cervical epithelial cells with *V. atypica* or *V. montpellierensis* induced significant elevation of metabolites associated with a higher cervicovaginal pH and amine odor, combined with depletion of metabolites involved with polyamine biosynthesis. *M. micronuciformis* infections significantly elevated glycerolipids and sphingolipids that are potentially indicative of cervical epithelial cell cytotoxicity (Fig. 5).

## Discussion

Members of the *Veillonellaceae* family, *V. atypica, V. montpellierensis,* and *M. micronuciformis* have been isolated from women with BV and healthy women^2,16,17,31,32,34,40^; however, the mechanisms employed by these bacteria that relate to BV pathogenesis and other gynecologic and obstetric sequalae are unknown. In this study, we aimed to elucidate the metabolic and inflammatory contributions of *V. atypica, V. montpellierensis,* and *M. micronuciformis* to reveal the function of these organisms in the cervical microenvironment.

To our knowledge, colonization of human organotypic genital epithelial cells by *V. atypica, V. montpellierensis,* or *M. micronuciformis* in vitro has not been previously demonstrated and visualized. Using our human 3-D cervical epithelial cell model, we demonstrated that *V. atypica, V. montpellierensis,* and *M. micronuciformis* are capable of colonizing cervical epithelial cell surfaces (Fig. 1B–D). Our human 3-D cervical epithelial cell model expresses mucins and TLRs and other physiological relevant features of parental human cervical tissue that may enhance colonization of bacteria compared to traditional 2-D monolayer cultures^46,52^.

The cytotoxic potential of *Veillonellaceae* members in the cervical epithelium has not been thoroughly explored. One study tested an unnamed *Veillonella* spp. on vaginal epithelial cell monolayers and found no evidence for cell death, though this was not quantified^53^. Our data suggest that *M. micronuciformis* induces moderate cytotoxicity, whereas *V. atypica* and *V. montpellierensis* do not significantly contribute to cervical epithelial cell death (Fig. 1). In this capacity, *M. micronuciformis* may therefore contribute to the breakdown of epithelial barrier function, in accordance with other BV-associated bacteria^4^.

To our knowledge, the pro-inflammatory host response to cervical infection with *M. micronuciformis* has not been previously examined in vitro, though another species under the *Megasphaera* genus (*Megasphaera elsdenii*) has been investigated^54^. In this study, dendritic cells elevated secretion of IL-1β, IL-6, IL-8, IL-12p40, and TNF-α in response to infection with *M. elsdenii*^54^. Additionally, *M. micronuciformis* was associated with genital inflammation as measured from cervicovaginal fluid in a cohort of South African adolescent females^55^. However, no studies have identified the immune response to *V. atypica* and *V. montpellierensis* in the cervicovaginal context. Our results suggest that *M. micronuciformis* induced a robust human 3-D cervical epithelial cell pro-inflammatory response marked by upregulated secretion of pro-inflammatory cytokines and chemokines, whereas both *V. atypica* and *V. montpellierensis* induced relatively fewer pro-inflammatory mediators (Fig. 2A). We hypothesize that *M. micronuciformis* is more pro-inflammatory than *V. atypica* or *V. montpellierensis* in the cervicovaginal environment, which is in the accordance with the literature, although more studies are required that investigate *Veillonella* spp. in the cervicovaginal environment.

SCFA production is a metabolic hallmark of BV as demonstrated in clinical and in vitro studies^56–58^ and have been linked to increased secretion of pro-inflammatory mediators^59^. We observed a higher accumulation of *N*-palmitoyl-sphingosine in response to infection with *M. micronuciformis* compared to *V. atypica* or *V. montpellierensis*, and is associated with inflammation^60^. *Veillonella* spp. and *M. micronuciformis* also induced upregulated secretion of several pro-inflammatory mediators from the 3-D cervical model, which is consistent with the literature and SCFA elevation^59^ and could indicate that SCFAs are both a hallmark of BV and indicators of cervical inflammation.

Histamine, a key metabolite involved in local host responses, was significantly accumulated by infection with *V. atypica* and *V. montpellierensis*. Other studies have found that histamine can decrease the expression of tight junctional proteins (ZO-1 in nasal epithelial cells and E-cadherin in epithelial pulmonary cells)^61^. Therefore, an increase in histamine may increase epithelial permeability, although this would need to be tested in the context of the cervicovaginal epithelium to confirm the role of histamine in barrier function within the cervicovaginal mucosa.

Our metabolomics analyses revealed changes in the lipid metabolism following infections with *Veillonellaceae* family members. Infection with *M. micronuciformis* induced significant accumulation in glycerolipids and sphingolipids (Fig. 5). We also observed a significant accumulation in glycerolipids and cholesterol during human 3-D cervical cell infections with *V. atypica, V. montpellierensis*, and *M. micronuciformis*. To date, there are few studies that have reported an association with elevated glycerolipids and cell death, but sphingolipids have been related to cell death^62^. In our previous clinical study, elevation in the relative abundance of sphingomyelin and 1-stearoyl-2-docosahexaenoyl-GPC correlated with *Lactobacillus* depletion and genital inflammation^51^. However, additional studies are needed in order to correlate elevated glycerolipids with cell death.

Early reports that dissect the metabolic potential of *Veillonella*^49,63–66^ revealed that *Veillonella* spp. utilize lactate as a sole energy source^49,64,67^. Health-associated *Lactobacillus* spp. secrete copious amounts of lactic acid into the cervicovaginal milieu^14,68,69^ and lactic acid is depleted in severe cases of BV^3^. It is therefore possible that *V. atypica* and *V. montpellierensis* elicit their pathogenicity in the early stages of BV when lactate is still available. Our data support lactate utilization by *V. atypica* and *V. montpellierensis* (Fig. 5). Since current literature indicates lactate consumption by other *Megasphaera* spp.^70^, our finding could be explained by suggesting that lactate serves as a non-primary energy source for *Megasphaera*. Importantly, we have shown that *Veillonella* spp. consume lactate (key metabolite associated with cervicovaginal health) and may contribute to changes in the cervicovaginal pH^71^. Indeed, in our previous clinical study investigating the vaginal microbiome in women across cervical neoplasia, *V. montpellierensis* and other *Veillonella* spp. were enriched in women with abnormal cervicovaginal pH^33^.

Elevation of cervicovaginal polyamines is a metabolic hallmark of BV and contributes to increased pH and amine odor, both of which are key clinical symptoms of BV^72^. Polyamine synthesis by *Veillonella* has been documented in the oral microbiome and contributes to malodor^73^. Additionally, one clinical study demonstrated that *M. micronuciformis* was associated with elevated cervicovaginal putrescine^14^. Our data revealed that in the context of our human 3-D cervical epithelial cell model, both *V. atypica* and *V. montpellierensis* modulate a variety of polyamines, including cadaverine, putrescine, histamine, and agmatine, whereas polyamines were not significantly elevated by *M. micronuciformis* infection (Fig. 5). Additionally, in previous clinical studies the metabolite 5′-methylthioadenosine (MTA), a by-product of polyamine synthesis, has been found to be depleted in women harboring non-*Lactobacillus* dominant microbiomes^72,74,75^. In our current study, MTA was also depleted in *V. atypica* and *V. montpellierensis* infections. An earlier study^14^ provided clinical correlations for putrescine production by *Megasphaera* in a vaginal context, which is not in accordance with our data; however, the next-generation sequencing method employed was unable to identify *Megasphaera* at the species level. It is possible that other *Megasphaera* spp. present in the cervicovaginal microbiome may exert differential metabolic capabilities that influenced the observed elevation of putrescine in this study^14^. However, both the increase in polyamine synthesis and depletion of MTA observed in infections with *V. atypica* and *V. montpellierensis* suggest that these microorganisms may significantly impact polyamine synthesis in vivo and influence metabolic hallmarks of BV (e.g., putrescine and cadaverine).

Our data also revealed arginine depletion following *M. micronuciformis* infection. Nitric oxide, agmatine, and ornithine are by-products of arginine catabolism^5,76^. Considering neither agmatine nor ornithine was significantly altered in our analysis, perhaps the observed depletion of arginine was a result of nitric oxide production by 3-D cervical cells. Nitric oxide has been implicated as an important toxic defense molecule against pathogens^77^ and has a role in inflammation and the immune response, further indicating pro-inflammatory properties of *M. micronuciformis* in the cervicovaginal microenvironment^78^.

**Fig. 6:**
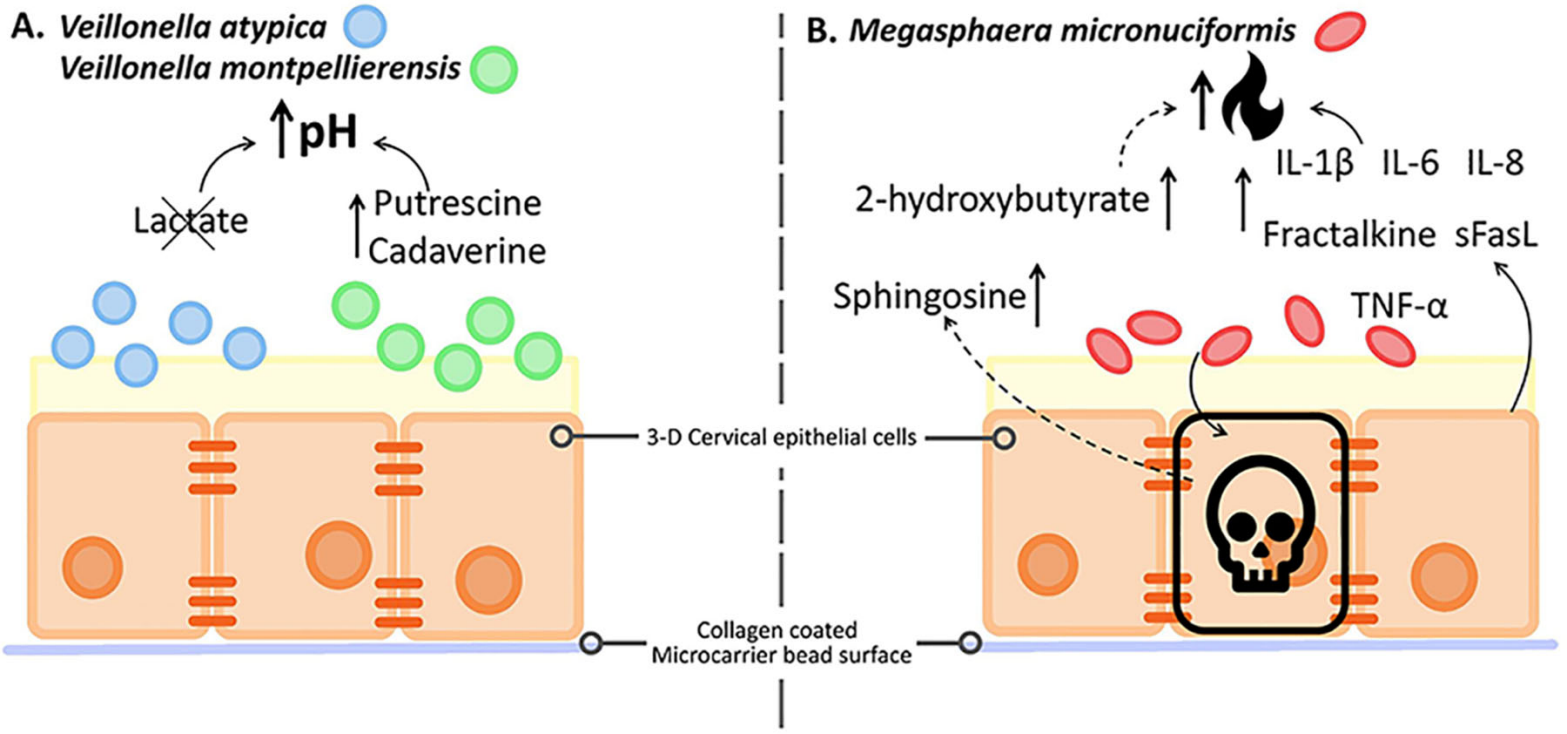
Members of the *Veillonellaceae* family uniquely modify the immunometabolic cervical microenvironment. Summary of immunoproteomics and metabolomics analyses of 3-D cervical cells infected with *V. atypica, V. montpellierensis* or *M. micronuciformis*. **A** Lactate was depleted and polyamines (putrescine and cadaverine) are significantly accumulated in cell culture supernatants of 3-D cervical cells infected with either *V. atypica* or *V. montpellierensis*. Lactate (metabolic hallmark of cervicovaginal health) depletion and elevated polyamine levels may correspond with an increase in pH, a clinical hallmark of BV. **B** The SCFA 2-hydroxybutyrate was significantly accumulated in the cell culture supernatants of 3-D cervical cells infected with *M. micronuciformis*. In addition, several pro-inflammatory mediators were elevated following infection with *M. micronuciformis*, including IL-1β, IL-6, IL-8, Fractalkine, sFasL, TNF-α. Finally, cytotoxicity/viability analyses revealed that *M. micronuciformis* infection resulted in moderate cervical epithelial cell death, as well as enrichment of sphingosine that could suggest the induction of cell death. Overall, *M. micronuciformis* may promote a pro-inflammatory immune profile within the cervical microenvironment, and cause cervical epithelial cell damage, compared to both *Veillonella* spp.

In summary, lactate depletion and polyamine production observed in our 3-D human cervical cell model suggest that *Veillonella* spp. contribute to elevated cervicovaginal pH via consumption of a key metabolite linked to gynecologic health (Fig. 6). We found that *M. micronuciformis* exhibited greater pro-inflammatory properties compared to *V. atypica* and *V. montpellierensis*. *M. micronuciformis* infections also resulted in higher levels of epithelial cytotoxicity and a higher enrichment in SCFAs that can also impact the production of pro-inflammatory cytokines and chemokines or cell death (Fig. 6). In line with our working hypothesis, our results provide evidence that *Veillonellaceae* family members alter the local microenvironment in a distinctive fashion. As putative primary colonizers, *Veillonella* spp. may create a favorable environment for secondary colonizers. On the other hand, *M. micronuciformis* may participate as a putative secondary colonizer that induces inflammation in the cervicovaginal tract and manifest as symptoms and clinical presentation of BV, as well as other gynecologic and reproductive sequalae (Fig. 6).

All experimental model systems have their own strengths and weaknesses^79^. We acknowledge the polymicrobial nature of BV and inter-bacterial and host-microbe interactions that participate in the development and progression of the disease. In this study, we used a reductionist approach and performed mono-infections using our robust 3-D cervical cell model to dissect the individual contributions of specific *Veillonellaceae* members. This approach is required to first understand individual pathologic contributions from these understudied bacteria. This data will provide the foundations for future studies that utilize polymicrobial cocktails that better represent the complexity and microbe-microbe interactions present in the microbiome. Finally, our untargeted metabolomics approach is beneficial since it allows us to examine metabolic changes on a global scale. This technique is limited, however, by the relativistic nature of metabolite measurements and is therefore unable to provide absolute metabolite quantification. Future studies employing targeted metabolomics will help to directly compare concentrations of key cervicovaginal metabolites within in vivo samples and in vitro models.

Future studies on *Veillonellaceae* members in the context of the cervicovaginal epithelium should focus on the temporal colonization patterns of BV-associated organisms, as this will provide insights into the potential contribution to biofilm formation for these microorganisms. In addition, studies of polymicrobial “cocktails” that combine *Veillonellaceae* members with other vaginal bacterial isolates will provide additional insights into bacteria-bacteria interactions in the context of the cervicovaginal microenvironment. In conclusion, the unique mechanisms and individual contributions of *Veillonellaceae* family members defined herein may promote our understanding of biofilm formation characteristic of BV and the underlying mechanisms that contribute to adverse obstetric/gynecologic health outcomes related to BV.

## Methods

### Human cervical epithelial cell monolayer and three-dimensional human cervical cell model culture

Human cervical epithelial cells (A2EN) were grown as monolayers with keratinocyte serum-free media (Fischer Scientific) supplemented with endothelial growth factor (5 ng/ml), bovine pituitary extract (50 µg/ml), and Primocin (100 µg/ml) in the humidified atmosphere of 5% CO_2_ at 37 °C^46^. Primocin was not included in the media used in the bacterial infection assays outlined below. For 2-D monolayer cultures, cells were seeded into culture-treated 24-well plates to a cell density of ~2 × 10^5^ cells/ml. Prior to seeding, cells were enumerated using the Countess automated cell counter (Invitrogen) and trypan blue exclusion, which were used for cytotoxicity assays.

To generate the 3-D cervical cell model, human cervical epithelial A2EN cells were grown on Cytodex-3® collagen-coated dextran microcarrier beads (Sigma-Aldrich) in a rotating wall vessel bioreactor (Synthecon), as previously described^15,45,46,80^. Following the 28-day differentiation period, aggregates were seeded into 24-well culture-treated plates at the density of 1 × 10^5^–5 × 10^5^ cells/ml and used for downstream analyses.

### Bacterial strains and growth conditions

*V. atypica* strain CMW7756B and *V. montpellierensis* strain DNF00314 were cultured on brain heart infusion agar (Becton Dickinson) supplemented with 5% (v/v) defibrinated sheep blood (Quad Five) and 0.56 mg/l of sodium d-lactate (Sigma-Aldrich). *M. micronuciformis* strain DNF00954 was cultured on tryptic soy agar (Becton Dickinson) supplemented with 5% (v/v) defibrinated sheep blood (Quad Five). Bacteria were grown at 37 °C under anaerobic conditions generated using AnaeroPack sachets and anaerobic environmental chambers (Thermo Scientific). All strains were obtained from Biodefense and Emerging Infections Research Resources Repository (https://www.beiresources.org). *M. micronuciformis* strain DNF00954 and *V. montpellierensis* strain DNF00314 were isolated from women with BV, whereas *V. atypica* strain CMW7756B was isolated from the genital tract of a pregnant woman.

### Bacterial infection of human cervical epithelial cells

Prior to the infection, bacteria were grown for 16–18 h on appropriate agar plates, resuspended in sterile Dulbecco’s phosphate-buffered saline (PBS), and adjusted to an optical density at 600 nm (OD_600_) 0.5. The viability of bacteria was confirmed using the standard plating assay. The adjusted bacterial cell suspensions were serially diluted in PBS, plated on appropriate agar media, and incubated for 96 h at 37 °C under anaerobic conditions for enumeration of colony-forming units (CFU). The bacterial suspensions of OD_600_ 0.5 corresponded to bacterial densities of approximately 1–2 × 10^8^ CFU/ml. Human cervical cell 2-D monolayers used for cytotoxicity assays were infected with a range of bacterial suspensions equivalent to OD_600_ of 0.001, 0.01, and 0.1 per 1× 10^5^ cervical cells/ml, which corresponded to multiplicities of infection (MOI) of 2–4, 20–40, and 200–400. Infected cervical cells were incubated for 24 h at 37 °C under anaerobic conditions. In a preliminary experiment, after 24 h incubation with cervical cells, the viability of bacteria was confirmed and did not change more than 1 log when compared to the initial inocula. The 3-D cervical epithelial cells were infected with the bacterial resuspensions at an MOI of 20–40 for 24 h at 37 °C under anaerobic conditions, as described above.

### Lactate dehydrogenase (LDH) assay to assess cytotoxicity

The LDH assay (Invitrogen, ThermoFisher Scientific) was performed according to the manufacturer’s guidelines. LDH activity was measured from cell culture supernatants by recording absorbance values at 490 and 680 nm. Spontaneous LDH activity was measured from PBS-treated cells and maximum LDH activity was measured from lysed cells. Percentage cytotoxicity was calculated by using the following formula: (bacteria-infected LDH activity − spontaneous LDH activity)/(the maximum LDH activity − spontaneous LDH activity) × 100.

### Scanning electron microscopy

Three-dimensional cervical epithelial cells were infected with each bacterial species at an MOI of 200–400 for 4 h at 37 °C under anaerobic conditions as described above. Samples were fixed and processed for scanning electron microscopy (SEM) as previously described^80,81^. Samples were imaged using a JSM-6300 JEOL scanning electron microscope and images were obtained with the IXRF model 500 digital processor (IXRF systems). SEM images were pseudo-colored using Photoshop 19.0 CC (Adobe).

### Immunoproteomic analysis of cell culture supernatants

Cell culture supernatants collected from at least three independent replicates of 3-D human cervical epithelial cells infected with bacteria as described above were used to quantify concentrations of 15 protein targets. PBS-treated cells served as negative controls. Cytometric bead arrays were performed using customized MILLIPLEX Multianalyte Profiling (MAP) Human Cytokine and Chemokine Panel 1, Human Circulating Cancer Panel 2, and Human Matrix Metalloproteinases Panel 2 (Millipore) in accordance with manufacturer guidelines. The tested targets included fractalkine, interleukin (IL)-1α, IL-1β, IL-6, IL-8, interferon-γ-induced protein-10 (IP-10), monocyte chemoattractant protein-1 (MCP-1), MCP-3, macrophage inflammatory protein-1β (MIP-1β), platelet-derived growth factor-AA (PDGF-AA), regulated on activation, normal T-cell expressed and secreted (RANTES), transforming growth factor-α (TGF-α), tumor necrosis factor-α (TNF-α), vascular endothelial growth factor (VEGF), macrophage migration inhibitory factor (MIF), tumor necrosis factor-related apoptosis-inducing ligand (TRAIL), soluble Fas ligand (sFasL), carcinoembryonic antigen (CEA), cancer antigen 125/mucin 16 (CA125), matrix metalloproteinase-1 (MMP-1), MMP-7, MMP-9, and MMP-10. Data was collected and analyzed using a Bio-Plex® 200 (Bio-Rad) platform and Bio-Plex® Manager (5.0) software (Bio-Rad). A 5-parameter logistic regression curve fit was used to determine concentrations. All reported values for MCP-1, MCP-3, MIP-1β, and CA125 were below the minimum detectable concentration and were therefore not included in the further analyses. All samples were assayed in duplicate.

### Untargeted global metabolomics analysis of cell culture supernatants

Cell culture supernatants collected from three independent 3-D cervical cell aggregate batches for both *Veillonella* strains and four independent batches for *M. micronuciformis* infections were sent to Metabolon Inc. (Durham, NC) for untargeted global metabolomics analysis. Metabolites were resolved on a Waters ACQUITY ultra-performance liquid chromatography (UPLC) and a Thermo Scientific Q-Exactive high resolution/accurate mass spectrometer interfaced with a heated electrospray ionization (HESI-II) source and Orbitrap mass analyzer operated at 35,000 mass resolution. The sample extract was dried then reconstituted in solvents compatible to each of the four methods. Each reconstitution solvent contained a series of standards at fixed concentrations to ensure injection and chromatographic consistency. One aliquot was analyzed using acidic positive ion conditions, chromatographically optimized for more hydrophilic compounds. In this method, the extract was gradient eluted from a C18 column (Waters UPLC BEH C18-2.1 × 100 mm, 1.7 µm) using water and methanol, containing 0.05% perfluoropentanoic acid (PFPA) and 0.1% formic acid (FA). Another aliquot was also analyzed using acidic positive ion conditions; however, it was chromatographically optimized for more hydrophobic compounds. In this method, the extract was gradient eluted from the same aforementioned C18 column using methanol, acetonitrile, water, 0.05% PFPA, and 0.01% FA and was operated at an overall higher organic content. Another aliquot was analyzed using basic negative ion optimized conditions using a separate dedicated C18 column. The basic extracts were gradient eluted from the column using methanol and water, however with 6.5 mM ammonium bicarbonate at pH 8. The fourth aliquot was analyzed via negative ionization following elution from a HILIC column (Waters UPLC BEH Amide 2.1 × 150 mm, 1.7 µm) using a gradient consisting of water and acetonitrile with 10 mM ammonium formate, pH 10.8. The MS analysis alternated between MS and data-dependent MS^n^ scans using dynamic exclusion. The scan range varied slighted between methods but covered 70–1000 *m*/*z*.

The bioinformatics system consisted of four major components, the Laboratory Information Management System (LIMS), the data extraction and peak-identification software, data processing tools for QC and compound identification, and a collection of information interpretation and visualization tools for use by data analysts. The hardware and software foundations for these informatics components were the LAN backbone, and a database server running Oracle 10.2.0.1 Enterprise Edition. Peaks were quantified using area-under-the-curve, which allows determining relative intensity of compounds among tested samples, but not the absolute concentrations.

### Statistical analyses

All infections and assays were performed as at least three independent replicates. One-way ANOVA with Dunnett’s adjustment for multiple comparisons was used to statistically analyze the immunoproteomics data. One-way ANOVA with Bonferroni post-hoc tests were used to test for significant differences in LDH data. Differences in metabolite pathway composition were determined by chi-squared analysis. One-way ANOVA analysis was performed using Prism v8 software (GraphPad). Hierarchical clustering analysis and heatmap visualization were performed using ClustVis^82^. Metabolite intensity values were median-scaled and log-transformed prior to performing two-tailed paired Student’s *t*-tests (infection vs. PBS control) using the R rstatix package. Metaboanalyst 4.0^83^ was used for principal component analysis (PCA), Pearson’s correlation analysis, and metabolite pathway enrichment analysis. *P*-values of <0.05 were considered significant in all analyses. Metabolomics results were corrected for multiple testing using the false discovery rate (FDR) and *q*-values were reported. All error bars represent standard deviation.

### Reporting summary

Further information on research design is available in the Nature Research Reporting Summary linked to this article.

## Supplementary information

Supplementary Information

Supplementary Data 1

Reporting Summary

## Data Availability

The authors declare that the data supporting the findings of this study are available within the paper and its Supplementary Information files. Additional data are available from the corresponding author upon reasonable request.
