# Supplementary material for: Veillonellaceae family members uniquely alter the cervical metabolic microenvironment in a human three-dimensional epithelial model
Source: NPJ Biofilms Microbiomes. 2021 Jul 6;7:57. doi: 10.1038/s41522-021-00229-0 (PMC8260719; doi:10.1038/s41522-021-00229-0)
Supplement: Supplementary file 1 — Supplementary Information [file 41522_2021_229_MOESM1_ESM.pdf]

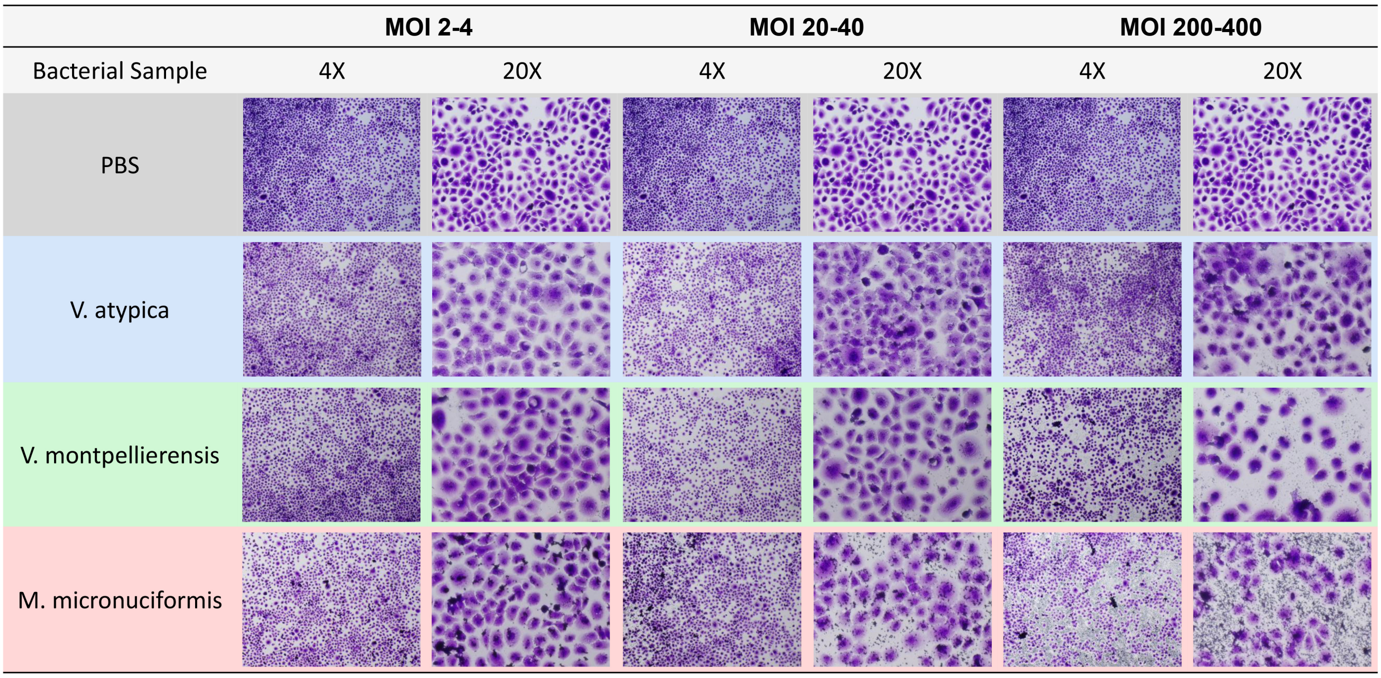

Supplementary Figure 1: *M. micronuciformis* monolayer infections induced moderate epithelial cell cytotoxicity, whereas *V. atypica* and *V.* montpellierensis induced low epithelial cytotoxicity. Monolayer cervical epithelial cells were infected with *V. atypica, V. montpellierensis,* and *M. micronuciformis* at MOI 2–4 , 20–40 and 200–400 for 24 hours at 37 °C under anaerobic conditions. Cells were washed with PBS and stained with crystal violet to visualize cytotoxity and morphological changes. Light microscopy at 4x and 20x magnification was used to visualize cell density differences at lower magnification and more detailed morphological changes at higher magnification.

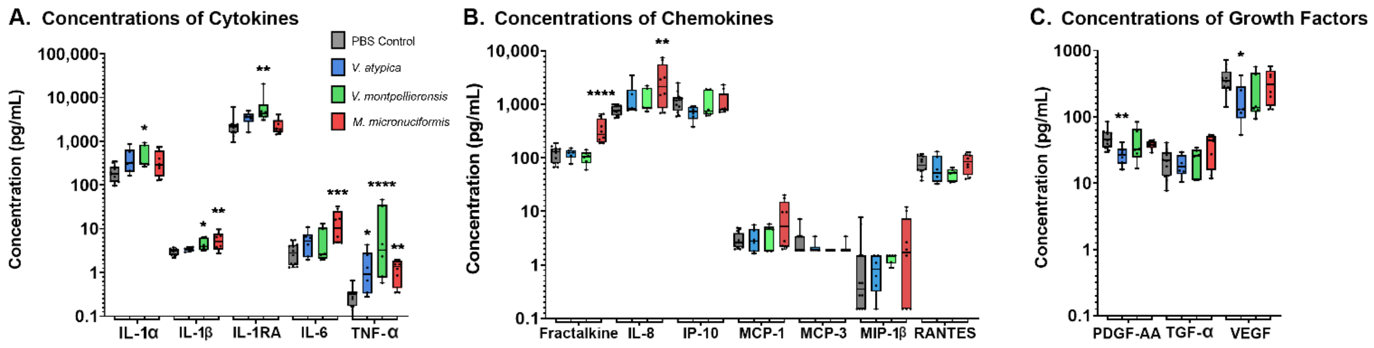

Supplementary Figure 2: *M. micronuciformis* induced elevated levels of pro-inflammatory immune mediators to a greater extent relative to *V. atypica* and *V. montpellierensis*. Immunoproteomics analysis was performed on collected cell culture supernatants following infection of 3-D cervical cells with either *V. atypica, V. montpellierensis* or *M. micronuciformis* for 24 hours at 37 °C under anaerobic conditions. Concentrations of (**A**) cytokines, (**B**) chemokines or (**C**) growth factors in cell culture supernatants were measured using Bio-Plex analysis. Data was transformed with $\log_{10}$, and statistically analyzed using one-way ANOVA with Dunnett's tests for multiple comparisons. Error bars represent standard deviation. Independent biological replicates were collected in triplicate for both *Veillonella* and in quadruplicate for *M. micronuciformis*. Two technical replicates were run for each independent biological replicate. *$P < 0.05$, **$P < 0.01$, ***$P < 0.001$, ****$P < 0.0001$.

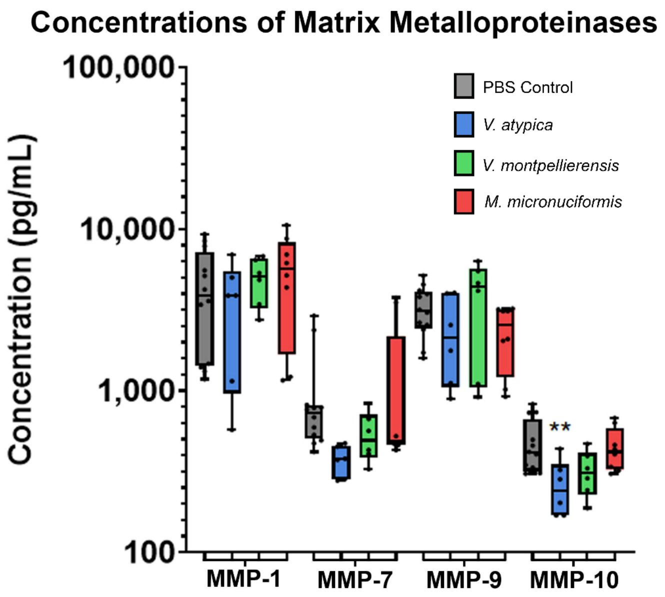

Supplementary Figure 3: *V. atypica* induces a significant decrease in MMP-10 concentration. Immunoproteomics analysis was performed on collected cell culture supernatants following infection of 3-D cervical cells with either *V. atypica, V. montpellierensis* or *M. micronuciformis* for 24 hours at 37 °C under anaerobic conditions. Concentrations of matrix metalloproteinases (MMPs) secreted into the cell culture supernatants were measured using Bio-Plex analysis. Only *V. atypica* induced a significant decrease in MMP-10 compared to PBS controls. Data was $\log_{10}$ transformed and statistically analyzed using one-way ANOVA analysis, with Dunnett's tests for multiple comparisons. Error bars represent standard deviation. Independent biological replicates were collected three times for both *Veillonella* and four times for *M. micronuciformis*. Two technical replicates were run for each independent biological replicate. ***P* < 0.01.

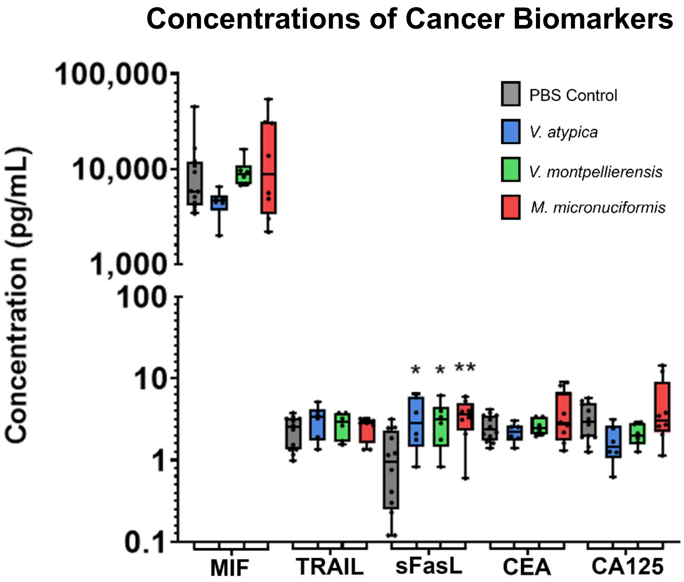

Supplementary Figure 4: *V. atypica, V. montpellierensis* and *M. micronuciformis* induce significant elevation of sFasL. Immunoproteomics analysis was performed on collected cell culture supernatants following infection of 3-D cervical cells with either *V. atypica, V. montpellierensis* or *M. micronuciformis* for 24 hours at 37 °C under anaerobic conditions. Concentrations of proteins that are associated with cancer and secreted into the cell culture supernatants were measured using Bio-Plex analysis. *V. atypica, V. montpellierensis* and *M. micronuciformis* induce significant elevation of sFasL. Data $\log_{10}$ transformed and statistically analyzed using one-way ANOVA with Dunnett's tests for multiple comparisons. Error bars represent standard deviation. Independent biological replicates were collected in triplicate for both *Veillonella* spp. and in quadruplicate for *M. micronuciformis*. Two technical replicates were run for each independent biological replicate. **P* < 0.05, ***P* < 0.01.

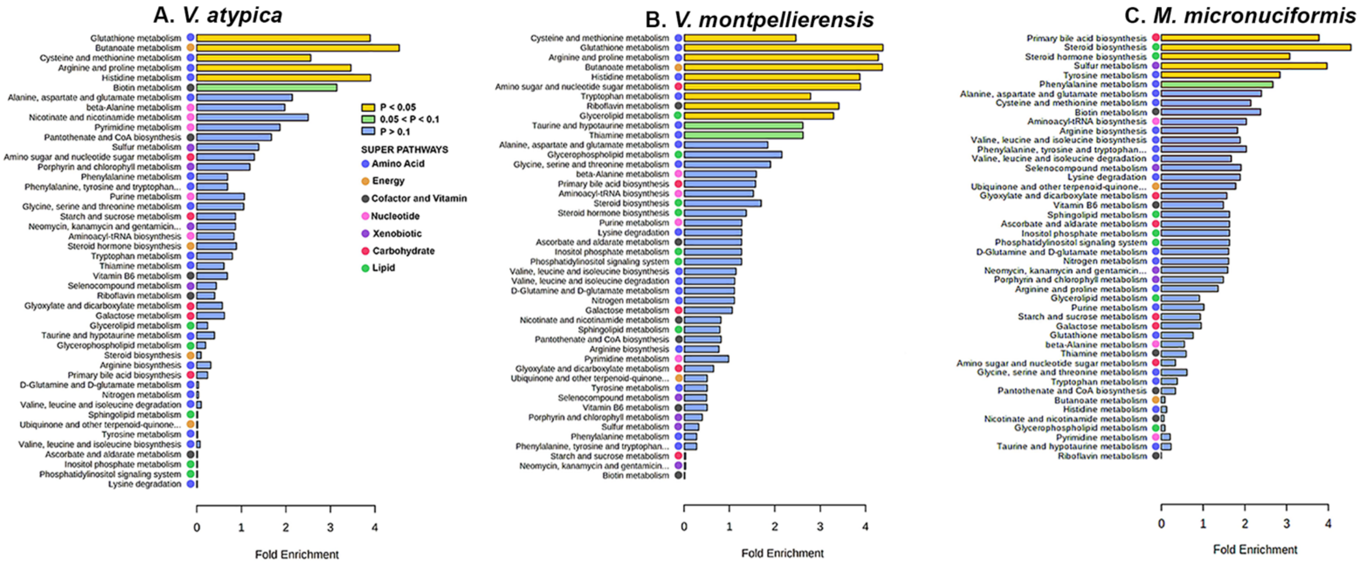

Supplementary Figure 5: Metabolic pathways enriched in 3-D cervical cell models infected with *V. atypica, V. montpellierensis* and *M. micronuciformis*. Metabolite pathway enrichment analysis of human 3-D cervical epithelial cell models infected with (**A**) *V. atypica,* (**B**) *V. montpellierensis* and (**C**) *M. micronuciformis* for 24 hours at 37 °C under anaerobic conditions. Yellow bars indicate significantly ($P < 0.05$) enriched pathways, green bars indicate pathways that approach significance ($0.05 < P < 0.1$), and blue bars indicate pathways that are not significantly enriched ($P > 0.1$). Pathways are color-coded with adjacent dots indicating their corresponding sub-pathways.
