# Supplementary material for: Veillonellaceae family members uniquely alter the cervical metabolic microenvironment in a human three-dimensional epithelial model
Source: NPJ Biofilms Microbiomes. 2021 Jul 6;7:57. doi: 10.1038/s41522-021-00229-0 (PMC8260719; doi:10.1038/s41522-021-00229-0)
Supplement: Supplementary file 3 — Reporting Summary [file 41522_2021_229_MOESM3_ESM.pdf]

nature research

Corresponding author(s): Dr Melissa Herbst-Kralovetz

Last updated by author(s): Apr 2, 2021

Reporting Summary

Nature Research wishes to improve the reproducibility of the work that we publish. This form provides structure for consistency and transparency in reporting. For further information on Nature Research policies, see our Editorial Policies and the Editorial Policy Checklist.

Statistics

For all statistical analyses, confirm that the following items are present in the figure legend, table legend, main text, or Methods section.

n/a	Confirmed	
☐	☒	The exact sample size (*n*) for each experimental group/condition, given as a discrete number and unit of measurement
☐	☒	A statement on whether measurements were taken from distinct samples or whether the same sample was measured repeatedly
☐	☒	The statistical test(s) used AND whether they are one- or two-sided<br>*Only common tests should be described solely by name; describe more complex techniques in the Methods section.*
☐	☒	A description of all covariates tested
☐	☒	A description of any assumptions or corrections, such as tests of normality and adjustment for multiple comparisons
☐	☒	A full description of the statistical parameters including central tendency (e.g. means) or other basic estimates (e.g. regression coefficient) AND variation (e.g. standard deviation) or associated estimates of uncertainty (e.g. confidence intervals)
☐	☒	For null hypothesis testing, the test statistic (e.g. *F*, *t*, *r*) with confidence intervals, effect sizes, degrees of freedom and *P* value noted<br>*Give P values as exact values whenever suitable.*
☒	☐	For Bayesian analysis, information on the choice of priors and Markov chain Monte Carlo settings
☐	☒	For hierarchical and complex designs, identification of the appropriate level for tests and full reporting of outcomes
☐	☒	Estimates of effect sizes (e.g. Cohen's *d*, Pearson's *r*), indicating how they were calculated

Our web collection on statistics for biologists contains articles on many of the points above.

Software and code

Policy information about availability of computer code

Data collection	Bio-Rad Bio-Plex Manager 5.0 software
Data analysis	1) Metaboanalyst 4.0: (https://www.metaboanalyst.ca/)<br>2) Metabolon Inc: (https://www.metabolon.com/)<br>3) R (version 3.6.3): (https://www.r-project.org/)<br>4) ClustVis: (https://biit.cs.ut.ee/clustvis/)<br>5) Prism v8 Software (GraphPad): (https://www.graphpad.com/scientific-software/prism/)

For manuscripts utilizing custom algorithms or software that are central to the research but not yet described in published literature, software must be made available to editors and reviewers. We strongly encourage code deposition in a community repository (e.g. GitHub). See the Nature Research guidelines for submitting code & software for further information.

Data

Policy information about availability of data

All manuscripts must include a data availability statement. This statement should provide the following information, where applicable:

- Accession codes, unique identifiers, or web links for publicly available datasets
- A list of figures that have associated raw data
- A description of any restrictions on data availability

The authors declare that the data supporting the findings of this study are available within the paper and its supplementary information files. Additional data are available from the corresponding author upon reasonable request.

Field-specific reporting

Please select the one below that is the best fit for your research. If you are not sure, read the appropriate sections before making your selection.

☒ Life sciences ☐ Behavioural & social sciences ☐ Ecological, evolutionary & environmental sciences

For a reference copy of the document with all sections, see nature.com/documents/nr-reporting-summary-flat.pdf

Life sciences study design

All studies must disclose on these points even when the disclosure is negative.

Sample size	Sample size was determined based on previous studies and the number of replicates needed to minimize variation and maximize statistical significance. For the immunoproteomics and metabolomics analysis we performed at least three independent batches of bioreactors run in duplicate for all analyses.
Data exclusions	No data was excluded from the analysis.
Replication	The data presented is representative of at least three independent replicates of the experiments.
Randomization	Cell culture samples were randomly evaluated for downstream analyses. However during the set up of each experiment, cell culture wells and experimental group were not randomized on the plate for the in vitro studies.
Blinding	The investigators performing the experiments were not blinded as that was not relevant to our study and would be difficult to complete.

Reporting for specific materials, systems and methods

We require information from authors about some types of materials, experimental systems and methods used in many studies. Here, indicate whether each material, system or method listed is relevant to your study. If you are not sure if a list item applies to your research, read the appropriate section before selecting a response.

Materials & experimental systems

n/a	Involved in the study	
☒	☐	Antibodies
☐	☒	Eukaryotic cell lines
☒	☐	Palaeontology and archaeology
☒	☐	Animals and other organisms
☒	☐	Human research participants
☒	☐	Clinical data
☒	☐	Dual use research of concern

Methods

n/a	Involved in the study	
☒	☐	ChIP-seq
☒	☐	Flow cytometry
☒	☐	MRI-based neuroimaging

Eukaryotic cell lines

Policy information about cell lines

Cell line source(s)	Human A2EN cervical epithelial cell line
Authentication	STR DNA profiling was performed and these cells are not contaminated with other cell lines found in available databases.
Mycoplasma contamination	The cell lines were not tested for mycoplasma contamination recently
Commonly misidentified lines (See ICLAC register)	The cell line used in this study is not commonly misidentified
